# An Evaluation of Ischaemic Preconditioning as a Method of Reducing Ischaemia Reperfusion Injury in Liver Surgery and Transplantation

**DOI:** 10.3390/jcm6070069

**Published:** 2017-07-14

**Authors:** Francis P. Robertson, Barry J. Fuller, Brian R. Davidson

**Affiliations:** 1 Division of Surgery and Interventional Science, Royal Free Campus, University College London, 9th Floor, Royal Free Hospital, Pond Street, London NW3 2QG, UK; b.fuller@ucl.ac.uk (B.J.F.); b.davidson@ucl.ac.uk (B.R.D.); 2 Department of Hepaticopancreatobiliary Surgery and Liver Transplantation, Royal Free Foundation Trust, 9th Floor, Royal Free Hospital, Pond Street, London NW3 2QG, UK

**Keywords:** Ischaemic Reperfusion injury, Ischaemic Preconditioning, Remote Ischaemic Preconditioning

## Abstract

Liver Ischaemia Reperfusion (IR) injury is a major cause of post-operative liver dysfunction, morbidity and mortality following liver resection surgery and transplantation. There are no proven therapies for IR injury in clinical practice and new approaches are required. Ischaemic Preconditioning (IPC) can be applied in both a direct and remote fashion and has been shown to ameliorate IR injury in small animal models. Its translation into clinical practice has been difficult, primarily by a lack of knowledge regarding the dominant protective mechanisms that it employs. A review of all current studies would suggest that IPC/RIPC relies on creating a small tissue injury resulting in the release of adenosine and l-arginine which act through the Adenosine receptors and the haem-oxygenase and endothelial nitric oxide synthase systems to reduce hepatocyte necrosis and improve the hepatic microcirculation post reperfusion. The next key step is to determine how long the stimulus requires to precondition humans to allow sufficient injury to occur to release the potential mediators. This would open the door to a new therapeutic chapter in this field.

## 1. Introduction

Ischaemia Reperfusion (IR) injury, the injury that happens to an organ when its blood supply is interrupted and re-established, is a major cause of post operative liver dysfunction, morbidity and mortality following liver transplantation and hepatic resectional surgery. Surgical and oncological advances in the treatment of colorectal liver metastases combined with an increase in obesity and aging populations in the West have led to an increase in major liver resectional surgery being performed on high risk patients. Similarly, the increased demand for liver transplantation combined with its proven success has led to a shortage of donor organs for transplant with the increased use of marginal quality grafts.

The presence of liver steatosis is associated with an increased risk of mortality from 2 to 14% following elective liver resection [1,2] and the use of a graft from a Donor following Circulatory Death (DCD) is associated with a twofold increase in risk of recipient death and graft loss [3]. This increased risk is secondary to the increased susceptibility of these livers to IR injury. There is currently no accepted treatment for IR injury and as such the development of strategies to ameliorate IR injury are necessary to make major liver surgery and liver transplantation safer. This would simultaneously allow the safe implantation of more marginal grafts that are currently rejected for transplantation due to the worry of Primary Graft Non Function (PGNF) resulting from severe IR injury.

Ischaemic Preconditioning (IPC), the process by which short bursts of ischaemia to a vascular bed results in protection during further sustained ischaemic periods, is one such strategy which is directly applicable in the clinical setting. IPC can be either applied directly to the target organ [4] or remotely to a distant vascular bed [5]. Both forms of IPC have been shown to successfully ameliorate IR injury in small animal models. However the translation to clinical practice has led to conflicting results following an initial positive trial by Clavien [6,7] on patients undergoing hepatic resection, there have been several negative trials and some positive trials [8]. A recent meta-analysis failed to identify any benefit resulting from IPC performed in liver resections [9] however a meta-analysis of IPC performed on transplant donor livers prior to graft retrieval found evidence of a reduction in post-operative recipient mortality and graft loss [10]. A key factor in these conflicting results is a lack of understanding of the mechanisms by which IPC exerts its protective effects and as such IPC/RIPC protocols vary between studies and are unlikely to be optimal. Furthermore, IR injury in the setting of humans is more complex than in small animal models as multiple pathways overlap and can be altered secondary to the underlying condition. In small animal models, the animals are healthy and have little genetic variation, IR injury happens in patients often with severe systemic disease and multiple co morbidities. It is well known in cardiac patients and small animal models that diabetes, obesity and increased aged reduce the beneficial effect of preconditioning [11] but the effect of chronic liver disease remains unknown. Further complicating factors include blood loss and the need for transfusion during major liver surgery, as potential humoral factors may be lost or diluted and the type of anaesthetic used. Recently, in the setting of cardiac surgery, two large randomized controlled trials have failed to demonstrate a benefit following RIPC with both trials identifying the use of intravenous propofol (a very common anaesthetic agent used regularly in liver resection and transplantation) as a potential limitation [12,13] and a small trial carried out by our group in the setting of liver transplantation [14] identifying that due to the use of high flow oxygen prevented the creation of true ischaemic conditions in the limb during the RIPC stimulus. As these essential intra-operative factors may impair the protection of RIPC knowledge of the various protective mechanisms is of key importance as this would not only allow IPC protocols in humans to be altered to overcome these hurdles but may also lead to new pathways that can be targeted to ameliorate IR injury bypassing these factors. The majority of basic research into IPC/RIPC has been performed in the myocardium. In this review, we present the mechanistic pathways identified in the protective effect of IPC/RIPC on liver IR injury.

## 2. Methods

Pubmed, Excerpta Medica Database (EMBASE) and Publicus Ovidus Naso (OVID) were searched between the years of 1986 and 2016 using the search strategy: (((liver) OR (hepatic)) AND ((ischemia) OR (ischemia-reperfusion injury)) AND ((preconditioning) OR (ischemic preconditioning) OR (IPC) OR (remote ischemic preconditioning) OR (RIPC) OR (hepatoprotection))). Key studies investigating the mechanisms of IPC/RIPC are contained in Table 1.

## 3. Protective Effects of Preconditioning

Although there is clear evidence in the setting of myocardial IR injury to suggest that IPC exerts its maximal effect in the immediate post reperfusion period [44], it is known that IPC has a bimodal duration of protection. There appears to be an early (or classic) window of protection lasting up to 3 h and a later period of protection lasting between 12 and 72 h post preconditioning—the so-called second window of protection (SWOP) [45,46,47]. The mechanisms by which IPC exerts it protection during these phases are poorly understood but have been shown to be very different in the two time periods. The phenomenon of the SWOP, although a focus of cardiac IPC studies has not been the focus of studies in hepatic IR injury, in which IPC/RIPC is generally performed 5 min before IR injury. This review will focus mainly on the classical protective window.

**Figure 1:**
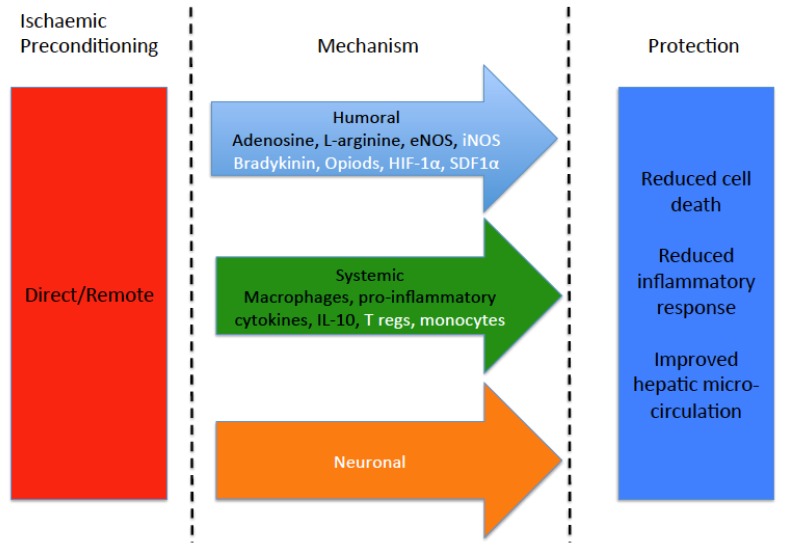
Previously identified mechanism of IPC. Mechanisms identified in the setting of liver IR injury are in black whilst those not implicated/researched are in white.

IPC has been postulated to work through three main generic mechanisms (Figure 1). IPC has been shown to release humoral factors into the blood which reduce apoptosis and cell death in the target organ during the IR injury. It has also been shown to reduce the systemic inflammatory response following IR injury further reducing tissue injury. It has been shown in other organs that RIPC relies on a neuronal feedback mechanism to provide protection and that interruption nervous system either physically or pharmacologically can block RIPC [48]. This pathway has not been investigated in the setting of liver IR injury.

## 4. Adenosine

**Figure 2:**
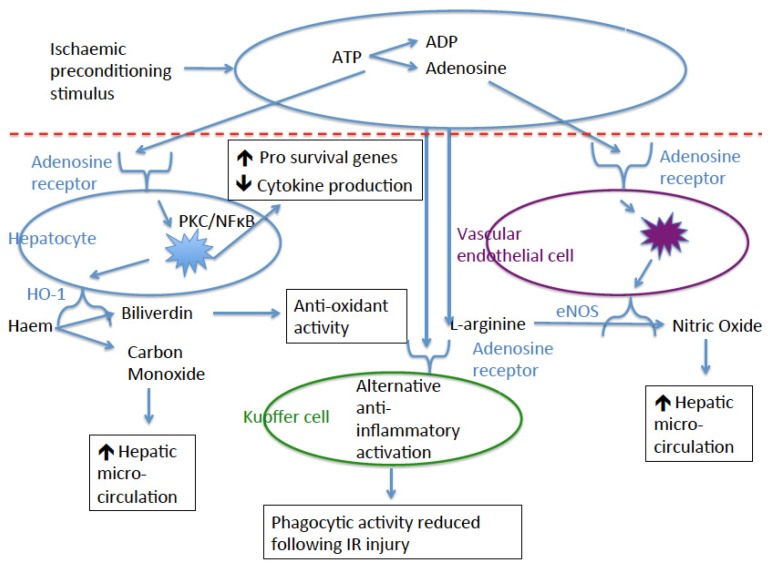
Protective mechanisms of adenosine release following Ischaemic Preconditioning (IPC).

Adenosine, a nucleotide and component of ATP breakdown, is rapidly released from damaged cells in ischaemic tissue [49] and can bind with four different adenosine receptors (A_1_R, A_2A_R, A_2B_R, and A_3_R) [50], all of which can be expressed by hepatocytes [51]. As in other organs [51], Adenosine has been shown to play a protective effect in the liver following IR injury through a diverse range of mechanisms (Figure 2). Pharmacological upregulation of endogenous adenosine by R75231 (which prevents adenosine uptake and metabolism) significantly attenuated liver IR injury in a canine model and led to significantly increased survival at two weeks following 2 h of total liver ischaemia [52]. In rats treated with adenosine deaminase (which degrades adenosine) prior to IPC, the protective effect of IPC was abolished in hepatic IR injury as measured by serum transaminases and lactate dehydrogenase [15]. Conversely, administration of a NO donor in these animal reinstated the protective effect which is in keeping with the knowledge that adenosine leads to NO release by the vascular endothelium, resulting in vasodilation [53]. The same group demonstrated that IPC increased levels of adenosine in the hepatic tissue [16] and this has been confirmed in one study [17]. The administration of adenosine deaminase abolished the protective effect of IPC and adenosine infusion prior to IR injury provided protection to a similar level as IPC [16]. An interesting observation by Peralta and colleagues was that 10–15 min of continuous ischaemic stimulus in IPC was the ideal length of time in rats as it lead to sufficient release of adenosine but insufficient release of other toxic metabolites of ischaemia [16]. This is in keeping with studies of IPC on donor livers prior to retrieval in human liver transplantation in which it was demonstrated that 5 min of donor preconditioning was insufficient to provide protection [54] whilst 10 min of donor IPC was associated with a reduction in postoperative transaminases in keeping with a reduction in IR injury [55], a finding also found in patients undergoing RIPC prior to major hepatectomy [8]. There is clear evidence that adenosine is upregulated following IPC/RIPC and that pharmacological degradation of adenosine ablated the protective effect of IPC. Adenosine exerts its anti-apoptotic and vasodilatary effect through its interaction with the adenosine receptors. In keeping with studies into IR injury in other organs, much of the work on these receptors has focused on the A_1_ and the A_2A_ receptors.

## 5. The A_1_ Receptor

Genetic knockout mice lacking the A_1_ receptor are more susceptible to hepatic IR injury than wild type mice with a normal A_1_R [56]. This was linked to a significant increase in the level of apoptosis and neutrophil infiltration seen within the liver at 12 and 24 h. Selective blockade of A_1_R with 8-Cyclopentyl-1, 3-dpropylxanthine (DPCPX) also increased IR injury as measured by serum transaminases and hepatic necrosis [56]. In contrast, beagles administered the A_1_R antagonist (KW3902) had reduced transaminases, better hepatic blood flow upon reperfusion and significantly improved two-week survival following hepatic IR injury (83% vs. 17%, *p* < 0.05) [57]. It is unclear why there is such a striking difference between the studies on the A_1_ receptor but this may reflect the different species or the use of a different A_1_R antagonist.

Adenosine or its receptors may also play a role in the protective effect of IPC. However some of the results have been conflicting. Ajemieh and colleagues [19] demonstrated that in rats treated with the A_1_R antagonist, DPCPX, the protective effect of IPC was ablated. Similarly treatment of rats with CCPA (an A_1_R agonist) provided a similar level of protection following IR injury as garnered by IPC. This was in contrast to findings from Peralta and colleagues [18] who demonstrated that, although adenosine depletion negated the protective effects of IPC and improved hepatic blood flow post reperfusion, pharmacological inhibition of the A_1_R with DPCPX did not affect the protection produced by IPC. The timing of DPCPX administration between these studies differs. It was administered 5 min prior to IPC by Perlata [18] and 24 h prior to IPC by Ajemieh and colleagues [19] which may explain the different results. Both studies used a dose of 0.1 mg/kg. In none of the above studies did treatment of the animals with an A_1_R agonist lead to protection of the liver during IR injury prompting the suggestion that IPC required the presence or upregulation of endogenous adenosine [19] or other mediators.

## 6. The A_2A_ Receptor

The A_2A_ Receptor has been shown to play a key role in hepatic IR injury as the administration of the A_2A_R agonist γ-glutamylcysteine synthase (GCS) to isolated rat livers immediately prior to reperfusion reduced the level of apoptosis and degree of liver IR injury as measured by transaminases and degree of hepatocyte apoptosis [58].

IPC has been shown indirectly to exert protection through the A_2A_R in several studies. Perlata and colleagues [18] demonstrated that the use of DMPX, an A_2_R antagonist (at this time point, there was no distinction between A_2A_R and A_2B_R), ablated the protective effect of IPC in a rat model. Thurman and colleagues [59] demonstrated again that the administration of DMPX ablated the protective effect of IPC but also they were one of few groups who demonstrated that the administration of CGS-21680, an A_2_R agonist, protected the liver against IR injury. Their results would suggest that IPC prevented sinusoidal epithelial cell death through the adenosine receptors. However, in contrast, Schaeur and colleagues [20] demonstrated that the use of DMPX had no effect on the protective effect of IPC. Adenosine has been shown to exert its protective effect through reduced hepatocyte apoptosis and increased hepatic blood flow post reperfusion. It has also been shown to play a role in directing the early immune response post reperfusion [60] and mice treated with an A_2A_R agonist (ATL146e) not only had a significantly reduced IR injury but also had less upregulation of pro-inflammatory cytokines including IL-6 and MCP-1. The activation of Natural Killer T cells was inhibited through activation of the A_2A_R [61] again suggesting that adenosine is able to suppress the post reperfusion inflammatory response. Whether this is as a result of reduced necrosis or directly suppressing inflammation remains to be elucidated as the effect of IPC on NKT cell differentiation and activation has not been investigated.

## 7. The A_2B_ Receptor

Few studies have investigated the role of the A_2B_R in hepatic IR injury. Zimmerman and colleagues [62] demonstrated that the A_2B_R was upregulated on human hepatocytes in post reperfusion liver biopsies when compared to the same livers pre implantation. Furthermore, they showed that mice lacking the A_2B_R suffered significantly worse IR injury following 45 min of warm IR injury and that this was associated with higher levels of IL-6 and TNFα production in the liver and distant end organs. Analysis using cell culture demonstrated that activation of the A_2B_R reduced NF-κB activation and stabilization in hepatocytes and that pharmacological stabilisation of NF-κB reconstituted the injury in A_2B_R deficient mice. In a study of global hypoxic preconditioning in which mice were subjected to 10% Oxygen for 10 min prior to hepatic IR injury, it was shown that mice lacking the A_2B_R were not protected while mice lacking each of the other three receptors were still protected [21]. Although reduction in IR injury was associated a reduction in IL-6, TNFα levels and neutrophil infiltration, no down stream mechanisms were explored.

## 8. The A_3_ Receptor

There is no evidence as yet regarding the role of the A_3_R either in hepatic IR injury or in IPC of the liver.

## 9. Adenosine and Its Receptors

The seemingly conflicting results from studies investigating the individual adenosine receptors would suggest that it is more likely that the mechanism of protection of IPC is related to increased adenosine release rather than upregulation of an individual receptor.

## 10. Nitric Oxide and Nitric Oxide Synthase

Nitric oxide (NO), a potent vasodilator, is a colourless gas synthesized by the action of Nitric Oxide Synthase (NOS) on l-arginine [63] and has been shown to exert a protective effect during hepatic IR injury [64] by inhibiting synthesis of endothelin, a potent vasoconstrictor [65]. There are three isoforms of NOS. Only two are believed to play a role in hepatic IR injury: endothelial nitric oxide syntase (eNOS) and inducible nitric oxide syntase (iNOS).

There is robust evidence demonstrating that NO derived from eNOS is hepatoprotective following IR injury. Transgenic mice lacking eNOS have been shown to suffer a more significant IR injury [66,67,68,69,70] whilst genetic over expression of eNOS in mice is associated with a significant reduction in IR injury [71].

In the setting of IPC, eNOS expression and circulating levels of l-arginine were upregulated in the rodent liver following IPC and this was associated with increased nitrate levels in the portal vein [22]. Pre treatment with DMPX, an adenosine A2 inhibitor, abolished the protective effect of IPC. There was no upregulation of eNOS and the results were similar to those seen when rats were treated with l-NAME, a NOS inhibitor, suggesting that eNOS upregulation and function is reliant on the A_2_R pathway [64]. These results were similar to the findings of Mathie and colleagues [72] who demonstrated that administration of adenosine was able to ameliorate IR injury but that when combined with l-NA, an eNOS inhibitor, adenosine was unable to provide protection [72]. Transgenic mice lacking eNOS that underwent RIPC displayed the same level of hepatic IR injury as those undergoing IR injury without RIPC again confirming the key role eNOS in the early protection of RIPC [23].

The effect of NO from iNOS in hepatic IR injury is more variable. Some studies have suggested that the effect of iNOS activation is dependent on the length of the ischaemic period and temperature maintained during ischaemia [63], however several studies have shown that NO derived from iNOS is a key mechanism of liver injury following IR injury [73,74]. Targeting eNOS rather than NO may therefore be more beneficial.

IPC of the liver has not been shown to affect iNOS levels [22] suggesting the iNOS does not play a role in the protective mechanisms of IPC/RIPC.

## 11. Protein Kinase C

The term protein kinase C (PKC) encompasses a family of intracellular enzymes that can be classified as signal transducers that direct the processing of downstream proteins. PKC induction in hepatocytes has been shown to be significantly elevated in rodent livers following reperfusion [75]. PKC has been identified as a downstream signalling pathway of adenosine receptors [76,77]. Pharmacological inhibition of PKC has been shown to reduce hepatic IR injury [26,78,79].

Interestingly, studies investigating the effect of IPC on PKC activity have shown that the protective effect of IPC is not associated with inhibition of PKC but actually with an increase in PKC activity. In an isolated hepatocyte model, it was shown that the protective effect of hypoxic preconditioning was ablated by the use of a PKC inhibitor chelerythrine [24]. Further similar work by the same group on isolated rodent hepatocytes confirmed these findings but also linked PKC activation to the A_2_R [77]. In pig livers undergoing IPC, prior to cold storage, PKC was shown to be activated in the hepatocytes of livers undergoing IPC prior to cold storage [26]. Treatment of livers with chelerythrine was shown to abolish the protective effects of IPC [26]. Other intracellular kinases have been implicated including tyrosine kinase [80], mitogen activated protein kinase [77]. Although PKC has been postulated as a potential mechanism for IPC, it would seem more likely that PKC and other intracellular kinases play a key role in indiscriminately transferring the extracellular signal generated by IPC/RIPC to the cell nucleus.

## 12. Nuclear Factor Kappa-Light-Chain-Enhancer of B Cells (NF-κB)

NF-κB is a transcription factor that is rapidly upregulated in ischaemic cells and has been shown to play a role in hepatic IR injury and to promote upregulation of iNOS and pro-inflammatory cytokines [75,81]. NF-κB levels were significantly upregulated within the first 4 h post IR injury in a murine model.

In mice that underwent IPC, NF-κB levels were significantly lower than those that did not [38]. This was associated with a reduction in TNFα mRNA levels. In porcine grafts undergoing IPC prior to cold storage, NF-κB translocation was upregulated early following IPC, prior to cold storage [27] suggesting that similar to PKC, NF-κB is more likely to be an intracellular messenger that is affected by extracellular molecules upregulated during IPC/RIPC.

## 13. Haem-Oxygenase-1 (HO-1)

Haem-oxygenase is an enzyme that catalyses the degradation of heme resulting in the production of anti-oxidant biliverdin and carbon monoxide [82] another gaseous signalling agent which has vasodilatory effects. Expression of HO-1 has been shown to be upregulated in hepatocytes following hepatic IR injury [28] and it has been shown to reduce hepatocyte apoptosis following IR injury, increase the availability of anti-oxidants, improve hepatic blood flow and to have anti-inflammatory effects [83] all of which have been suggested may ameliorate IR injury. Treatment of mice with gadolinium choride has been shown to upregulate HO-1 expression of Kupffer cells promoting an anti-inflammatory phenotype in Kupffer cells that was absent in HO-1 genetic knock out mice, and was associated with reduced liver injury [84]. Pharmacological upregulation of HO-1 with isoproterenol has been shown to reduce cytokine release from macrophage cell culture via down regulation of NF-κB following lipopolysaccharide stimulation and to reduce HMGB-1 release (a key driver of liver IR injury). This was associated with a significantly increased seven-day survival from 30 to 70% in a rat model of peritonitis [85]. Similar findings have been shown in a rat model of cardiac IR injury, in which again treatment with isoproterenol prior to occlusion of the left anterior descending artery for 30 min significantly increased circulating levels of HO-1 and HO-1 activity which was associated with significantly reduced levels of IL-6, TNFα and HMGB1 and significantly reduced infarct size [86]. Transduction experiments of rat livers with a viral vector for HO-1 injected into the portal vein prior to graft harvest have shown upregulation of HO-1 up to 90 days post transplantation and is associated with increased survival and immune tolerance [87] as demonstrated by an increased level of Tregs, Il-10 and TGF-β in both the liver and the periphery.

RIPC of the hind limb has been shown to significantly upregulate HO-1 expression and activity on hepatocytes. This was measured after 4 h but prior to IR injury and was associated with a significant reduction in IR injury as measured by transaminases [28]. In contrast at 2 h post direct IPC and IR injury, although IR injury was ameliorated and HO-1 mRNA levels were upregulated, HO-1 was not detectable on hepatocytes, but was by 24 h, suggesting that upregulation of HO-1 takes several hours and may not be the earliest protective mechanism of IPC [29]. Interestingly, although HO-1 was upregulated on hepatocytes, there was no change identified in circulating lymphocytes [28]. The same group demonstrated in mice that HO-1 expression is upregulated following IR injury suggesting that the upregulation of HO-1 is necessary pre IR injury to be effective [30]. Upregulation of HO-1 in the liver following RIPC was seen to increase the incidence of autophagy [30]. This is the process by which cells envelop and degrade damaged cellular components within the cytoplasm preventing them from leaking in to the surrounding extracellular space where they act as damage associated molecular patterns (DAMPs) and may explain why RIPC can be associated with a reduction in HMGB1 release following IR injury [86].

## 14. The Immune System

IR injury is an example of sterile inflammation. Following reperfusion, the release of DAMPS into the extra cellular space provokes an intense inflammatory activation and several cell types have been implicated in this process.

## 15. CD4+ T Cells

CD4+ T cells are a component of the lymphocyte populations and are rapidly recruited to post ischaemic tissue. There is clear evidence that CD4+ T cells play a key role in IR injury as mice lacking CD4+ T cells, although suffering a similar ischaemic injury, are protected from the reperfusion injury [74]. This phenomenon has not only been shown in the murine liver, but is evident in the murine kidney [88], and in the murine lung [89]. The CD4+ T cell population is composed of both pro inflammatory effector T cells and anti-inflammatory T cells. The effect of IPC on effector T cells has not been investigated as research has focused on the anti-inflammatory subgroup—regulatory T cells (Tregs). Three studies have looked at the effect of IPC on Treg recruitment and function, two in the kidney [31,32] and one in the liver [33]. The study protocols for the two studies in the kidney were very similar and IPC was performed seven days prior to the IR injury. Both studies demonstrated similar findings that not only did IPC ameliorate renal IR injury but also that Treg numbers in the ischaemic kidneys were upregulated. This upregulation of Tregs was not seen when the IR injury was performed three days or 14 days post IPC, suggesting that it is a delayed and transient phenomenon [31]. Treg function was measured by IL-10 production as measured by intracellular flow cytometry and this was significantly upregulated by IPC. Antibody depletion of Tregs by anti-CD25 antibody [32] or PC61 [31] ablated the protective effect of IPC. Furthermore adoptive transfer of Tregs obtained from mice that underwent IPC provided protection in naive mice [32]. In contrast, when IPC was performed immediately prior to IR injury, despite protection during IR injury, there was no evidence of Treg mobilization to the liver [33]. Furthermore depletion of Tregs did not ablate the protective effect of IPC and transfer of pre-activated Tregs into mice did not protect against IR injury and the authors came to the conclusion that the protection gained by IPC is independent of Tregs. The experiment they failed to perform was to augment Tregs into preconditioned mice and to add Tregs from preconditioned mice into naive mice. IPC has been shown to result in a reduction in circulating cytokines especially IL-6 which is known to act as a brake of Treg activation, proliferation and function [90] and to promote CD4+ effector T cell migration [91]. It is most likely that key factor in the differences between these studies is the timing between the preconditioning stimulus and IR injury. It is perhaps unrealistic to expect IPC to have a profound effect of T cells populations within such a short period of time. However, IPC/RIPC is more likely to alter the cytokine milieu that may affect early T cell responses and direct the later T cell response.

## 16. Macrophages

Kupffer Cells are resident liver macrophages and have been shown to be activated early following IR injury [92,93]. However experiments blocking macrophage activity have had varied results with some studies demonstrating that Kupffer cell blockade or modulation attenuated IR injury [94,95] whilst other models have shown increased IR injury following Kupffer cell depletion [96].

IPC has been shown to reduce Kupffer cell activation following IR injury as measured by reduced phagocytosis of latex particles [36] reduced reactive oxygen species secretion [34] and reduced TNFα secretion leading not only to reduced hepatic IR injury [34] but also reduced neutrophil accumulation in distant end organs [35]. However, arguably the most important study is a study from Tejima and colleagues in which the results suggest that IPC directly reduced hepatocycyte injury and death rather than through suppressing Kupffer cell activation [37]. Interestingly though they found in the absence of Kupffer cells, this protection was not gained suggesting a key role for macrophages in the preconditioning stimulus. This is in keeping with the theory that IPC/RIPC works by causing limited tissue injury resulting in protective mechanisms being activated.

## 17. Monocytes

Inflammatory monocytes are rapidly recruited to sites of tissue injury from the bone marrow by chemokine ligand 2 (CCL2) [97]. Few studies have looked at the role of inflammatory monocytes in hepatic IR injury, however they have been shown to play a key role in acetaminophen liver injury in small animal models [98]. One study using genetic CCL2 knock out mice has demonstrated that these are protected from hepatic IR injury leading to the suggestion that inflammatory monocytes play a key role in the pathogenesis of IR injury [99]. No studies have investigated the effect of IPC/RIPC on inflammatory monocytes.

## 18. Cytokines

Hepatic IR injury is associated with the early release of several pro-inflammatory cytokines. IL-2 [100], IL-6 [100,101], IL-17 [102], and TNFα [100,101] have been shown to be upregulated following hepatic IR injury and to be associated with increased hepatocyte apoptosis and neutrophil infiltration into the post ischaemic liver. TNFα is a key pro inflammatory cytokine that has been shown to play a role in hepatic IR injury and treatment of mice with anti-TNFα prior to IR injury is associated with a significant reduction in injury [100].

Serum cytokines have been measured in many small animal models of IPC/RIPC and there is strong evidence to show that IPC and RIPC are associated with a reduction in TNFα production in the liver in the first few hours following reperfusion [39,43]. Pre-treatment of mice undergoing IPC with DPCPX, an A_1_R antagonist abolished the protective effect of IPC and the reduction in serum TNFα levels suggesting that adenosine signalling may dampen down the immune response [19]. Furthermore, inhibition of NF-κB translocation by IPC led to a reduction TNFα mRNA in murine livers following IR injury [38].

IL-6 levels have similarly been shown to be reduced in the early hours post IR injury in mice undergoing IPC [40,42]. However, IPC is associated with a spike in IL-6 levels within the first hour post IR injury [42]. Although no studies of IPC/RIPC in hepatic IR injury have been performed in either IL-6 or TNFα deficient mice, studies have suggested that both IL-6 and TNFα are essential for hepatic regeneration and studies of hepatectomy in IL-6 deficient mice have demonstrated a more significant IR injury with increased mortality [40,103,104]. It should be noted that this model purposefully used a small for size liver remnant and several studies have shown that IPC is protective in normal livers. Similar results have been seen with TNF receptor deficient mice [105]. This may be explained by the fact that some immune cells have been shown to be pleiotropic and inflammatory monocytes recruited to sites of sterile inflammation in the liver were seen to phenotypically change and become alternative monocytes which are essential to tissue regeneration [106].

There has been much interest in IL-10, a potent anti-inflammatory cytokine both in terms of whether its supplementation may ameliorate IR injury and whether it is upregulated by IPC/RIPC. Treatment of mice with recombinant IL-10 prior to IR injury significantly attenuated IR injury and interestingly was associated with a reduction in TNFα production in the liver [105]. IL-10 depletion has been shown to result in increased liver injury and increased production of TNFα and IL-6 (89) [95] again demonstrating an interplay between these pro-inflammatory and anti-inflammatory cytokines and suggesting that manipulating cytokine production could alter IR injury. The effect of IPC on IL-10 levels post IR injury have been conflicting. In a rodent model, of direct RIPC, although IR injury was attenuated, IL-10 levels were not upregulated [42]. In contrast, in a trial of donor IPC in human liver transplantation, recipients that received a liver that underwent IPC prior to retrieval has significantly higher serum levels of Il-10 at three hours post reperfusion [41]. This was not associated with a reduction in IR injury but was associated with a significant reduction in both moderate and severe acute rejection episodes.

Although IL-6, IL-10 and TNFα knockout mice do exist as do neutralizing antibodies, these experiment have not to our knowledge been performed and as such, although IPC has been shown to alter levels of these cytokines, it remains unknown as to whether this is necessary for the protection garnered by IPC or as a consequence of IPC reducing hepatocyte death.

## 19. Conclusions

IPC/RIPC are an inexpensive and easily applied mechanism depending on intrinsic survival responses for protection during IR injury. Despite being first described in 1986 [4] and 1996 [5], the mechanisms by which they provide protection remain unclear and this has hampered their clinical translation. The release of adenosine from the ischaemic tissue during the preconditioning stimulus would appear to be the earliest and potentially the initial mechanism for signalling protection. Work from Peralta and colleagues [16] demonstrates that the optimal length of the preconditioning stimulus requires a delicate balance between being long enough to release sufficient quantities of adenosine but not too long that it simultaneously releases toxic metabolites. No studies have been done to date in humans to investigate the optimal time required for the preconditioning stimulus however studies measuring adenosine release in the organ undergoing the preconditioning stimulus may shed light on this. Following adenosine release and stimulation of the various adenosine receptors, there is evidence of decreased apoptosis and enhanced autophagy reducing release of DAMPS. Intracellular signalling via protein kinases and NF-κB leads to upregulation of pro survival pathways including HO-1. ENOS is upregulated along with l-arginine levels and the resulting NO produced has been shown to improve hepatic microcirculation and reduce the reperfusion injury. It is difficult to identify the predominant method of protection in these small animal studies as each study has only investigated one pathway and blockade/genetic knockdown of that pathway has abrogated the protective effect of IPC/RIPC. In humans, the picture is more complicated by the interplay of all of the pathways and the effect of chronic disease on these pathways. The next key step is to measure how long the IPC/RIPC stimulus requires to be applied in humans to release adequate adenosine/l-arginine as this may allow IPC/RIPC to be more successfully translated to clinical practice.

## Figures and Tables

**Table 1:** Studies investigating the mechanism of Ischaemic Preconditioning (IPC)/ Ischaemia Reperfusion Preconditioning (RIPC) in the setting of hepatic IR injury.

Study Group	Year	Species	IPC Time (min)	Ischaemic Time (min)	Reperfusion Time (min)	Hepatic Ischaemia	Pharmacological Manipulations	Parameters Assessed	Outcome of IPC	Proposed Mechanism
**Adenosine**										
Peralta [15]	1997	rat	10	90	90	partial	Adenosine and NO	LFTs	↓ LFTs	Adenosine/NO
								Hepatic blood flow	↑ blood flow	
Peralta [16]	1998	rat	Variable	90	90	partial	Adenosine and NO	LFTs Adenosine Inosine Xanthine	↓ LFTs ↑ Adenosine	Adenosine
Nakayama [17]	1999	rat	10	45	Up to 7 days	unclear	A_1_ and A_2_ receptors	LFTs	↑ 7 day survival	Adenosine via A_2_ receptor
								7 day survival	↓ LFTs	
								Adenosine	↑ Adenosine	
**A_1_ receptor**										
Peralta [18]	1999	rat	10	90	90	partial	A_1_, A_2_ receptors and NO	LFTs	↓ LFTs	NO production through action of adenosine on A_2_R
								Hepatic blood flow	↑ blood flow	
								NO production	↑ NO production	
Ajamieh [19]	2008	rat	10	90	24 h	partial	A_1_ receptor	LFTs	↓ LFTs	A_1_ receptor
								TNFα levels	↓ TNFα levels	
								MPO activity	↓ oxidative stress	
**A_2A_ receptor**										
Perlata [18]	1999	rat	10	90	90	partial	A_1_, A_2_ receptors and NO	LFTs	↓ LFTs	NO production through action of adenosine on A_2_R
								Hepatic blood flow	↑ blood flow	
								NO production	↑ NO production	
Schaeur [20]	2003	rat	10	90	120	partial	A_2A_ receptor and p38 MAPK	LFTs	↓ LFTs	p38 MAPK stimulation not A_2A_ receptor
								Hepatic perfusion	↓ KC induce liver damage	
**A_2B_ receptor**										
Chouker [21]	2012	mouse	10	45	240	partial	A_2A_, A_2B_ receptors	LFTs	↓ LFTs	A_2B_ receptor but not A_2A_ receptor
								TNFα levels	↓ TNFα levels	
								IL-6 levels	↓ IL-6 levels	
**A_3_ receptor**										
None										
**eNOS**										
Koti [22]	2005	rat	5	45	120	partial	l-arginine and NO	LFTs	↓ LFTs	NO formed from eNOS is hepatoprotective
								NO	↑ NO levels	
								eNOS	↑ eNOS	
								iNOS	no change in iNOS	
Abu-Amara [23]	2011	mouse	4	40	120	partial	eNOS genetic knockout	LFTs Hepatic blood flow Pathological injurye NOS expression	↓ LFTs ↓ injury eNOS expression not upregulated in wild type mice.	RIPC provided no protection in eNOS−/− mice
										RIPC did not upregulate eNOS expression in wild type mice
**iNOS**										
Koti [22]	2005	rat	5	45	120	partial	l-arginine and NO	LFTs	↓ LFTs	NO formed from eNOS is hepatoprotective
								NO	↑ NO levels	
								eNOS	↑ eNOS	
								iNOS	no change in iNOS	
**PKC**										
Carini [24]	2000	rat	10	90	0	hepatocytes	PKC	Intracellular pH	↑ cell survival	PKC necessary to allow IPC
								Intracellular Na	↓ pH	
								Cell viability	↓ Na accumulation	
Carini [25]	2001	rat	10	90	90	hepatocytes	A_2A_ receptor and PKC	Cell viability	↑ cell survival	PKC activation following A_2A_ receptor stimulation
								PK levels	↑ p38 MAPK phosphorylation	
Ricciardi [26]	2001	pig	15	120	240	total	PKC	Graft function	↑ Graft function	PKC translocation to nucleus is necessary for IPC
								Hepatic perfusion	↑ Hepatic perfusion	
								Graft injury	↓ Graft injury	
**NF-κB**										
Ricciardi [27]	2002	pig	15	120	240	total	PKC	NF-κB	↑NF-κB	IPC increases translocation of NF-κB
**HO-1**										
Lai [28]	2006	rat	10	45	240	partial	HO-1	LFTs	↓ LFTs	RIPC increases HO-1 expression and activity
								HO-1 expression	↑ HO-1 expression	
								HO activity	↑ HO activity	
Datta [29]	2014	mouse	5	45	120	partial	eNOS genetic knockout	LFTs Hepatic perfusion HO-1 expression	↓ LFTs ↑ Hepatic perfusion	eNOS−/− mice had reduced effect from IPC. HO-1 mRNA no significantly increased by IPC
Wang [30]	2014	mouse	4	45	24 h	partial	HO-1	LFTs	↓ LFTs	RIPC lead to increased autophagy in a HO-1 dependant manner
								HO-1 expression	↑ HO-1 expression	
								Autophagy	↑ Autophagy	
**Tregs**										
Kinsey [31]	2010	mouse	24 (bilateral)	28 (7 days post IPC)	unclear	Renal (1 kidney)	Treg depletion and adoptive transfer	Serum creatinine Renal Treg number and IL-10 production	↓ Creatinine ↑ Treg accumulation ↑ Treg IL-10 production	Treg accumulation took 7 days. Treg depletion ablated effect of IPC
Cho [32]	2010	mouse	24 (bilateral)	28 (7 days post IPC)	24 h	Renal (1 kidney)	Treg depletion and adoptive transfer	Serum creatinine	↓ Creatinine	Treg depletion ablated effect of IPC. Stimulated lymphocytes from mice undergoing IPC were less pro-inflammatory.
								Treg number	↑ Treg accumulation	
								Splenocytes cytokine and proliferation	↓ Splenocyte proliferation and cytokine production	
Devey [33]	2012	mouse	15	50	24 h	partial	Treg depletion and adoptive transfer	LFTs Treg numbers Circulating cytokines	↓ LFTs Treg recruitment	IPC mechanism not related to Tregs
**Macrophages**										
Peralta [34]	1999	rat	10	90	90	partial	TNFα treatment and macrophage depletion with Gadolinium Chloride.	LFTs	↓ LFTs	TNFα production by macrophages drives hepatic IR injury
								Hepatic oedema	↓ TNFα release	
								TNFα release	↓ hepatic oedema	
Peralta [35]	2001	rat	19	90	90	partial	Antibody inhibition of I-CAM and macrophage depletion with Gadolinium Chloride	LFTs Neutrophil accumulation and activity in distant organs	↓ neutrophil accumulation and activity in distant end organs	IPC reduce neutrophil infiltration into distant organs but not the liver itself. Likely secondary to macrophage TNFα production
Glanemann [36]	2003	rat	5	45	90	global	Nil	LFTs	↓ LFTs	IPC reduction macrophage activation in early staged of IR injury
								Kupffer cell phagocytosis	↓ Kupffer cell activation	
								Hepatic perfusion and oxygenation	↑ hepatic perfusion and oxygenation	
Tejima [37]	2004	rat	10	40	60	partial	Macrophage depletion with Gadolinium Chloride and treatment with anti-oxidants	LFTs	↓ LFTs	Macrophages were essential for the preconditioning stimulus to be effective
								Sinusoidal epithelial cell injury	no change in sinusoidal epithelial cell injury	
**Cytokines**										
Funaki [38]	2002	mouse	15	70	240	global	NF-κB an tyrosine kinase inhibition	Hepatic TNFα	↓ TNFα	IPC reduced hepatic TNFα levels
Zhu [39]	2003	rat	10	240 (cold)	24 h	global	nil	LFTs	↓ LFTs	IPC lead to reduced apoptosis and TNFα release
								Serum TNFα	↓ TNFα	
								Apoptosis	↓ Apoptosis	
Yao [40]	2007	rat	10	55	7 days	global	nil	Survival	No change in survival	IPC increased IR injury in small for size grafts
								LFTs	↑ LFTs	
								Hepatic IL-6	No change in TNFα	
								Hepatic TNFα	↓ IL-6	
Koneru [41]	2007	human	10	329-505	n/a	global	nil	Survival LFTs Post-op complications Serum TNFα Serum IL-6 Serum IL-10	No change in survival ↑ LFTs in the first 2 days ↓ episodes of acute rejection ↑ IL-10 levels post reperfusion No change in TNFα or IL-6 levels	
Ajamieh [19]	2008	rat	10	90	24 h	partial	A_1_ receptor	LFTs	↓ LFTs	A_1_ receptor
								TNFα levels	↓ TNFα levels	
								MPO activity	↓ oxidative stress	
Guimaraes [42]	2015	rat	4	45	180	partial	nil	LFTs Serum IL-6 Serum IL-10	↓ LFTs ↑ IL-6 at 1 h ↓ IL-6 at 3 h	IL-6 levels were raised 1 h post IPC but were significantly less at 3 h
Li [43]	2016	mouse	10	120 (cold)	3 days	global	nil	Survival	No change in survival	IPC reduced liver injury but did not improve survival
								LFTs	↓ LFTs	
								Serum TNFα	↓ TNFα	
								Innate immune response	↓ Apoptosis	

Abbreviations used: eNOS (endothelial Nitric Oxide Synthase), HO-1 (Heam-oxygenase-1)iNOS (inducible Nitric Oxide Syntase), KC (Kupffer Cells), LFT’s (Liver Function Tests), MAPK (Mitogen-activated Protein Kinases), MPO (Myeloperoxidase), NF-κB (Nuclear Factor kappa-light-chain-enhancer-of B cells), NO (Nitric Oxide), PK (Protein Kinase) TNFα (Tumour Necrosis Factor alpha), Tregs (regulatory T cells).
